# Supplementary material for: Are carbon emissions trading and green financial instruments synergistic? -Comprehensive quantitative research based on content analysis
Source: PLoS One. 2024 Mar 7;19(3):e0298601. doi: 10.1371/journal.pone.0298601 (PMC10919601; doi:10.1371/journal.pone.0298601)
Supplement: S1 Data — (ZIP) [file pone.0298601.s001.zip › Data/地级市Z_F工作报告.zip!/┴╔─■/╕╖╨┬╩╨/└┤╫╘╣1⁄2╓┌║┼í╛┬φ┐╦╩2╛▌═°í┐_2014.docx]

各位代表：

　　现在，我代表阜新市人民政府向大会报告工作，请予审议，并请市政协各位委员和其他列席同志提出意见。

　　一、2013 年工作回顾

　　过去一年，面对严峻复杂的经济形势，在市委的正确领导下，在市人大、市政协的监督支持下，我们团结带领全市各族人民，集中精力发展经济，全力推进社会事业，倾心尽力改善民生，实现了经济社会较快发展，奠定了突破阜新坚实基础，迈出了转型振兴坚实步伐。

　　预计完成地区生产总值620亿元，增长10%；公共财政预算收入70.5亿元，增长10.1%；固定资产投资572亿元，增长15%；社会消费品零售总额230亿元，增长13.5%；城镇居民人均可支配收入19370 元，增长13.1%；农村居民人均纯收入9915元，增长13%。全市主要经济指标增幅高于全省平均水平。

　　——产业集群建设加快。十大产业集群新开工项目168个；实现销售收入845亿元，增长35%；实现税金16.6亿元。

　　大唐煤制天然气项目一期第一系列全线调试；液压产业瀚石钻机等18个整机项目竣工；氟化工、皮革产业销售收入增长40%，玛瑙产业增长30%；板材家居、新型材料产业销售收入达到60亿元；铸造产业销售收入超百亿元。北京国能煤焦油、中国航天液压、江苏氟托、富新皮革、沈阳君航等项目开工，伊利乳业一期、新益达羊毛加工、晟宇铸造二期等项目竣工投产，与徐工、凯莱英、天士力、中国机械等集团合作加深。氟化工、皮革、铸造、新型材料产业研发中心及皮革交易中心等项目进展顺利。国家新能源示范市获批，新增风电装机容量7.5万千瓦。

　　开展“项目建设年”活动，新开工投资五千万元以上项目330个，其中亿元以上项目150个。

　　——三次产业稳步发展。净增规模以上工业企业80户，达到650 户，在全省位次前移；新增亿元以上企业20户、10亿元以上企业2户；实现倍增企业159户。阜矿集团成为中国企业500强。阜棉异地搬迁投入运营，远东重组、大金提质改造等项目进展顺利。新增高新技术企业11户、省级以上工程技术研究中心4家；高新技术产业增加值65亿元，增长35.4%。民营经济增加值增长32.9%。

　　农业喜获丰收，粮食产量55亿斤，创历史最好水平。沈阜200万亩现代农业示范带全面启动。实施“畜牧业强市”战略，新建、改扩建规模养殖小区312个，畜牧业标准化水平居全省第二位。完成节水滴灌工程40.3万亩。农田综合机械化水平达到82.5%，居全省前列。农村土地综合整治30万亩。

　　中央商务区等5个服务业集聚区营业收入230?亿元；温泉旅游度假区年接待能力20万人次；十家子玛瑙、开发区汽车贸易两个集聚区晋升省级行列。新增服务业限额以上企业100户。宝地太阳广场、浙商大厦开工，兴隆CBD、亚欧国际建材市场主体竣工，五金建材城、世通物流园投入运营。金融机构新增贷款95.6亿元；交通银行阜新分行、阜新银行营口分行开业运营，广发银行阜新分行获批筹建。荣获“中国玛瑙之都”称号。海棠山、大清沟被评为省级生态旅游示范区。

　　——招商引资成效显著。坚持高层推动招商，赴长三角、珠三角、港台、美韩等国家和地区招商44 次，签约落地投资亿元以上项目76 个，实现合资合作项目83个。工业地产招商取得突破，重庆盈田等10 个项目落地开工。全市招商引资500亿元，增长23.5%；直接利用外资2.05亿美元，增长12%。

　　成功举办“九市一盟”区域合作峰会、第八届中国·阜新玛瑙博览会、皮革展销会等活动。沈阜互动发展得到深化。成功晋升国家级高新区。

　　新增出口超五百万美元企业4 户。全年出口总额2.3亿美元，增长21.7%。

　　——城市功能明显提升。玉龙新城新增开发面积50万平方米，红星国际广场等项目开工，六合嘉际等项目主体完工，龙城广场、水系、道路、管网等配套工程扎实推进。彰武县被列为省新型城镇化建设试点县。

　　京沈客专阜新段征地拆迁全面展开。巴新铁路新邱至赤峰大板段铺架完成；阜盘高速及北延伸线通车，高速阜新站扩容完成，城市大外环、皮革园区快速通道建成；拓宽改造城市道路28条；建设维修农村道路545公里。新建及改造城区地下管网277公里。

　　房地产业投资119 亿元，增长30%；商品房销售额68 亿元，增长15%。建筑业总产值270亿元，增长23.7%，增幅居全省前列。

　　深入开展市容环境综合整治活动，城区垃圾一级清运机械化率达到30%，主城区营运人力三轮车全部退出，电动三轮车限行管理，市容环境进一步改观。

　　——生态环境持续改善。全年植树102万亩，完成200万亩经济林工程；实现退坡地还林6210亩，清理小开荒还林2086亩，围栏封育里程1686 公里；草原沙化治理23 万亩。省级以上公益林补偿面积363.4万亩。

　　开发区、清源和氟化工、皮革基地污水处理厂稳定达标运行，新增城市污水日处理能力10万吨。11 座乡镇污水处理设施建成。城区自备水源井封闭工作全面展开。

　　海州区氟化工企业、细河区铸造企业搬迁全面启动，海州露天矿环境治理工程实施，煤炭物流园前期工作、辽西危废处置中心建设进展顺利。国家循环经济试点市验收合格。环境空气质量二级以上天数达到95.3%。

　　城区植树147 万株，新增绿地137 万平方米。农村环境整治全面展开，145 个行政村治理达标。

　　—— 社会民生全面改善。重点民生工程全部完成。我市成为全国三个扶贫改革试验区之一。实现城镇实名制就业6.3 万人。城乡低保、农村五保、重点优抚对象的生活补助标准提高10%以上。企业退休职工基本养老金月人均增加136 元。新农合筹资标准每人每年提高60 元。职工最低工资标准每月提高120 元。保障性安居工程开工15671 套。接管77 处住宅供水二次加压泵房，城区自来水实现了从源头到用户的全程监管。建设公益性墓地14 座。新建改造公厕17 座，沿街单位内厕对外开放75 座。

　　辽宁工程技术大学国家大学科技园投入使用。与东北大学等7 家高校院所开展全面合作。建设标准化学校50 所，新建、改扩建幼儿园12 所，改造维修校舍5 万平方米。建成7 个县区数字图书馆和35 个乡镇电子阅览室。《三字经外传》在央视播出。圆满完成“ 十二运”分赛场赛事。成功创建国家“全民健身示范城市”。市第二医院儿科综合楼主体完工，阜矿总医院内科病房楼和第四、第五医院住院大楼、老年活动中心投入使用。阜蒙县被评为省级卫生县城。

　　—— 政府自身建设得到加强。自觉接受市人大依法监督和市政协民主监督，办结人大代表建议163 件、政协委员提案189 件。开展“ 工作创新年”和“规范管理年”活动，制定了转变职能简政放权等48 项制度措施，促进了政府效能提升、依法行政；出台了企业做强做大、企业整体搬迁、房地产业发展、工业地产招商等一系列政策，优化了发展环境；取消和调整行政审批事项53 项，将20 项经济管理权限下放城区，释放了县区发展活力；30 个乡镇行政服务中心建成使用，实现了全覆盖。全国第三次经济普查全面展开。“ 五大系统”建设深入推进，公共资源交易平台投入使用。安全生产、信访稳定、社会治安形势总体良好。十一届村委会换届圆满完成。民族地区繁荣发展，宗教领域和谐稳定。双拥共建成果显著，军政军民团结更加巩固。

　　司法、计生、审计、统计、质监、工商、人防、民防、食品药品监督、物价、地震、地方志等工作取得新成绩，外事侨务、对台事务、青年事务、妇女儿童、老龄、残疾人、慈善、红十字会和国地税、气象、电力、通讯、保险工作开创新局面，各行各业、各条战线呈现了新变化，为阜新经济社会发展作出了重要贡献。

　　各位代表，过去一年，我们聚精会神、全力以赴推进了一批重大项目、完成了一批重点工程、解决了一批难点问题、办成了一批惠民实事，巩固了发展形势，奠定了发展基础。

　　这些成绩的取得，是省委、省政府大力支持的结果，是市委正确领导的结果，是市人大、市政协监督支持的结果，是全市人民共同奋斗的结果。在此，我代表市人民政府，向人大代表、政协委员，向各民主党派、工商联、无党派人士和各人民团体，向省直单位、驻阜部队、武警和消防官兵，向所有参与、关心和支持阜新发展的同志们、朋友们，表示衷心的感谢和崇高的敬意！

　　在总结工作的同时，我们也清醒地认识到全市经济社会发展中存在的困难和问题。主要是：经济总量小、产业结构性矛盾尚未根本改变，多数产业集群还没有真正形成支撑能力；项目引进和储备不足，特别是缺少牵动力强的大项目，企业合资合作步子不大；服务业新兴业态特别是品牌项目少，县区服务业集聚区尚未真正形成；农业规模化、集约化水平不高，农业现代化步伐需要加快；土地、环保、资金等要素制约不断加大，生态基础薄弱的状况还未根本改变；个别单位、人员服务意识差、办事效率低、工作质量不高，软环境建设有待加强。对于这些问题，我们将在今后的工作中认真加以解决。

　　二、2014 年工作安排

　　当前，国内经济形势趋稳向好，发达地区产业转移大势所趋，国家出台了《全国资源型城市可持续发展规划》、实施了新型城镇化规划，辽宁省委、省政府深入实施突破辽西北战略，市委、市政府作出了新一轮突破阜新全面部署，我们面临新目标、新机遇、新支持，必须增强责任感、使命感、紧迫感，切实强化发展意识、实干意识、创新意识，尽职尽责，奋发有为，全力推动突破发展。

　　今年政府工作的总体要求是：深入贯彻落实党的十八届三中全会精神，按照市委十一届五次全会部署，紧紧围绕“尽快做大总量、努力提高质量”总要求，坚持以发展为第一要务，以结构调整为主线，以改革创新为动力，以项目建设为核心，以工业产业集群、服务业集聚区、现代农业示范带、新型城镇化建设为重点，努力推进经济发展、产业升级、社会进步、生态文明、民生改善，向转型振兴宏伟目标全力迈进。

　　主要预期目标是：地区生产总值700 亿元，增长10%；公共财政预算收入78.2 亿元，增长11%；固定资产投资685 亿元，增长20%；直接利用外资2.46 亿美元，增长20%；出口总额2.76 亿美元，增长20%；社会消费品零售总额261 亿元，增长13.5%；城镇居民人均可支配收入21300 元，增长10%；农村居民人均纯收入11000 元，增长11%。主要经济指标增幅仍将高于全省平均水平。

　　新一年的政府工作，必须以提高经济发展质量和效益为中心，必须以做大经济总量、做强财力、提高人民生活水平为根本，必须以稳中求进、稳中求优、稳中突破为基调，必须以改革创新为引领，必须以招商引资为突破，着力抓产业、抓项目、抓运行、保民生，高标准、高效率、高质量地完成全年各项工作。

　　（一）锲而不舍招商引资，全力推进项目建设

　　开展“ 招商引资突破年”活动，全年招商引资750 亿元，增长50%；新开工投资五千万元以上项目260 个，其中亿元以上项目100 个；竣工投产项目200 个。

　　实现各产业招商突破，重点引进国内外500 强、上市公司、行业领军企业和核心技术。十大产业集群引进资金增长50% 以上，煤化工、液压、氟化工、皮革、板材家居五个重点产业集群固定资产投资均超过50 亿元；全市引进投资10 亿元以上项目10 个，合资合作项目100 个；引进投资亿元以上现代农业项目30 个、现代服务业项目20 个；各县区至少新开工投资10 亿元以上项目1 个、工业地产项目1 个。

　　创新招商机制，突出长三角、珠三角、京津冀和港台等重点地区，深入开展驻地招商、自主招商、产业转移承接招商。坚持领导带头，推进外出招商制度化、常态化。充分借助商会、行业协会的力量，大力开展以商招商、专业招商。加大工业地产引进力度，依托工业地产联合招商。坚持务实、高效原则，提高招商引资针对性和实效性。制定出台优惠政策，支持各类企业挂靠联合、合资合作。加强与沈阳的互动发展，强化与周边地区的交流合作。

　　推进液压、氟化工等产业争创省级出口基地，新增有外贸出口实绩企业20 户。

　　建立重大项目跟踪包保制度，把项目开工率、当年投资额度作为考核的重要内容，促进项目加快落地、开工、投达产。做好用地保障、环评把关、金融支持、审批提速等工作，根据项目投资额度和工程进度，优先保障要素供给。包装储备一批重大项目，提高项目质量。

　　（二）聚精会神推进产业集群发展，做强做大骨干企业

　　加快十大产业集群建设，煤化工产业力争实现销售收入50 亿元，液压产业实现450 亿元，氟化工、皮革、板材家居产业力争达到100 亿元，铸造产业实现140 亿元，农产品、新型材料、玛瑙产业实现30%以上增长。

　　煤化工产业向精细化、系列化发展，大唐煤制天然气一期第一系列投产，开工建设二、三系列及鑫河LNG 等项目，推进大唐煤制天然气二期、新疆天业煤制乙二醇等项目前期工作。液压产业向整机化、高端化、品牌化发展，新开工项目40 个，深化与徐工、中国航天等集团合作。氟化工产业向精细化发展，开工建设凯莱英医药、恒通润滑油等15 个项目。皮革产业向制成品发展，入驻企业达到100 户以上。板材家居产业向整个家居行业延伸，新开工项目15 个。

　　铸造产业向机械加工、整机生产发展。新能源装机容量新增30 万千瓦。新型材料基地开工北京圣火产业园等32 个项目。玛瑙产业新开工十家子玛瑙城、玛瑙博物馆等项目。

　　铸造基地污水处理厂及氟化工、皮革、铸造、新型材料产业研发中心投入使用。规划建设商贸物流平台等配套项目。

　　实施企业倍增计划，工业销售收入突破千亿元。支持企业并购重组、开拓市场、地工产品协作，新增规模以上企业70 户、亿元以上企业20 户、10 亿元以上企业2 户，销售收入增长50% 以上企业超过100 户。阜矿集团本埠总收入突破150 亿元。

　　实施重大技改项目40 个，推动德尔扩能、大金提质改造、太克搬迁等项目投产。开工建设上海宝钢轻型钢合作、振 隆募投升级等项目，筹备建设中石化炼油厂，加快天士力健康产业园项目进程。

　　鼓励民间资本进入实体经济，扶持个体户转型为企业，新增小微企业300 户，民营经济增加值增长25%以上。

　　（三）切实加快集聚区建设，促进服务业提质升级

　　培育壮大中央商务区、温泉旅游度假区等4 个省级现代服务业集聚区，促进各城区特色服务业集聚区尽快形成。开工建设紫金财富广场、兴隆CBD 二期、万隆大厦、花园市场改造等项目，加快建设红星国际广场、恒强滨湖国际等项目，重点推进亚欧国际建材市场、宝地太阳广场、浙商大厦等项目运营。新增限额以上企业50 户。

　　加快商贸流通体系建设，开工建设五金建材城二期、世通物流园二期、中农农资大市场等项目，豪德贸易广场一期主体完工。

　　优化房地产开发结构，扩大商品房销售。实现建筑业总产值300 亿元，增长15%。

　　抓住建设全国旅游扶贫试验市机遇，打造海棠山文化名山，加快瑞应寺、大清沟、关山敖包文化旅游开发，促进温泉消费和旅游，培育乡村游等热点。推进葛根苏木旅游度假区规划建设。

　　组织开展银企对接，充分发挥金融服务企业、推动发展作用。金融机构新增贷款90 亿元。积极引进股份制及城市商业银行，推动广发银行阜新分行、农村商业银行运营。组建创投公司1 家，实现2 户企业在主板上市、5 户企业在“新三板”上市。促进小额贷款公司及担保行业健康发展。

　　（四）突出现代农业示范带建设，夯实富民强县基础

　　推进沈阜200 万亩现代农业示范带高起点开局，建成果品林示范区18 万亩、设施农业区15 万亩、精品农业示范区18 万亩、高效农业区4.5 万亩，阜彰两县各建成一个农产品专业市场。

　　深入实施“ 畜牧业强市”战略，新建改造高标准、规范化养殖小区50 个，培育壮大畜牧业市场。

　　加快辉山乳品加工、伊利乳业二期、雨润等重大农产品加工项目建设，开工建设北京红螺等5 个亿元以上项目。

　　继续做大做强阜蒙县花生“一县一业”品牌，争创省级示范县；尽快培育彰武县畜牧业特色优势。

　　完成节水滴灌工程77 万亩。推广各类农机具1 万台套。做好人工影响天气工作。

　　（五）积极推进新型城镇化，提高城乡建设管理水平

　　科学编制《阜新市新型城镇化发展总体规划》，完成《阜新市城市总体规划》修编。进一步提升玉龙、温泉、沈彰等新城规划档次和水平，修编县区总体规划和小城镇规划，实现城乡规划全覆盖。

　　强化政策驱动，制定我市新型城镇化发展实施意见，围绕集聚产业、企业和人口，研究产城互动、农民有序转化市民、公共服务设施配套、多渠道融资等政策措施。

　　统筹新型城镇化发展速度、质量、效益和布局，全年实施城镇开发面积450 万平方米，城镇化率提高3.5 个百分点。

　　玉龙新城核心区全面开工，一期20 平方公里基础设施全面完工，新增开发面积150 万平方米。实施旧城更新地块55 个、170 万平方米；抓住国家新一轮棚户区改造机遇，推进10 个城中村、城边村改造。继续推进“ 三位一体”规划，加快主城区、阜蒙县城、新邱区连接带建设。全力做好沈彰开发大道和柳河治理工程，促进沈彰新城与彰武县城有效衔接。推进城区6 个近郊镇全域城镇化，抓好阜彰两县18 个重点镇建设，打造一批特色中心镇、示范镇。

　　开展“ 城市综合整治年”活动，做好老旧小区改造、基础设施完善、环境提升等重点工程，抓好城市保洁、交通秩序、环境扰民等集中整治，建立专业化、属地化城市管理体制，强化“ 门前三包”、市民参与等制度保障，打造整洁、文明、美丽的城市环境。继续实施“五化工程”，新植大树18 万株，新增绿地面积55 万平方米；主要街路垃圾一级清运机械化率达到90%；提升19 条街路照明水平；对13 条人行道升级改造。

　　（六）加强生态环境和基础设施建设，打造美丽宜居阜新

　　继续抓好青山碧水蓝天工程，突出环境整治重点，全面完成海州区氟化工企业和细河区铸造企业搬迁；发电企业脱硫除尘设施稳定运行；做好海州露天矿环境治理工程；城区自备水源井实现彻底关闭；辽西危废处置中心投入使用；加快煤炭物流园建设；巩固电动三轮车管理成果。

　　实施细河流域治理，启动5 个生态净水工程、4 个生态示范点、3 个生态示范区建设，力争河流水质达标，细河城市段建成10 公里绿化景观带。

　　巩固和发展流域治理成果，污水处理设施全部稳定运行。做好九营子河、高林台河生态治理。

　　继续开展农村环境整治工程，14 个乡镇、157 个行政村治理达标。

　　抓好200 万亩经济林补植补造、“两退一围”管护、封山禁牧等工作，全年营造林86 万亩。草原沙化治理24 万亩。

　　推进气化阜新规划实施，积极发展清洁能源。重点做好燃煤企业监管、空气质量监测和预警系统建设等工作，环境空气质量二级以上天数达到95%以上。

　　推进国家“ 双百工程”示范基地建设，提高煤矸石、粉煤灰、矿井水、煤层气的综合利用水平，培育循环经济试点企业10 户，关闭小煤矿15 家。万元地区生产总值能耗下降3.9 个百分点。

　　全力做好京沈客专征地拆迁工作，配合做好工程建设；巴新铁路全线通车，做好与赤大白铁路在大板的联接线建设。加快阜盘铁路、阜奈高速前期工作。推进辽西北供水阜新配套工程和500 千伏输变电工程。

　　（七）统筹社会事业发展，提升公共服务能力

　　加快发展高新技术产业，培育科技创新示范企业80 户，创建省级以上工程技术研究中心30 个，完成重点科技创新项目40 项。支持辽宁工程技术大学重点学科建设和高端人才培养，深化与上海有机所等高校院所合作。

　　重视紧缺型、高层次、适用型、技能型人才的培养和引进，积极搭建企业、农村人才平台。做好大学生志愿服务辽西北工作。

　　促进各级各类教育均衡发展，推进标准化学校和基础教育强县区建设。优化职业教育资源，建设一职专国家级中等教育改革示范校。改善民族学校办学条件。深化课堂教学改革，大力发展素质教育。抓好师德师风建设。

　　积极打造文化品牌，加快特色文化产业发展，提升玛瑙博览会档次和水平。广泛开展群众文化活动，继续推进文化惠民工程。繁荣文化创作，做好辽宁省第九届艺术节准备。基本完成市区有线电视数字化整体转换。市图书馆对市民开放。

　　丰富健身活动，巩固“ 全民健身示范城市”成果；创新篮球赛事，扩大篮球城影响；积极备战省运会，提高竞技体育水平。

　　推进市妇女儿童活动中心、残疾人康复中心投入使用，开工建设市传染病院和蒙医院异地新建及卫校实训楼等项目。

　　完善社会治安防控体系，严密防范和依法打击违法犯罪活动。健全群众诉求表达、社会矛盾调解机制，加大信访问题化解力度。强化安全生产监管，提高防范预警和应急处置能力，坚决遏制重大事故发生。全面提高食品药品安全监管水平。支持民族特色产业和文化发展。发挥好宗教在促进社会和谐方面的积极作用。争创第八轮双拥模范城。

　　（八）保障改善民生，努力让人民生活更幸福

　　新增城镇实名制就业5 万人，培育技能人才2000 人，提高高校毕业生就业率。开展创业型县区、街道（乡镇）创建活动，加快创业孵化基地建设。

　　进一步扩大社会保险，促进应参尽参、应保尽保，新型农村和城镇居民社会养老保险参保率、持续缴费率均达到90%以上。

　　实施保障性安居工程1 万套以上。继续实施“蓓蕾生命救助行动”等救助项目。健全妇女儿童、老龄、残疾人等关爱服务体系和保障制度。着力构建和谐劳动关系。

　　继续推进公益性墓地建设，新建公墓23 座。

　　与此同时，我们切实抓好一批惠民便民实事。

　　1、改造完善老旧小区85 个、386 万平方米，打造安全、整洁、宜居的生活环境。

　　2、现有住宅节能改造100 万平方米。

　　3、建设改造城区地下管网80 公里；发展集中供热面积200 万平方米、煤气用户8000 户。

　　4、改造城市街路9 条，整治小街小巷9 条。

　　5、新建、改造农村公路200 公里。

　　6、解决农村2.2 万人饮水安全问题。

　　7、完成农村危房改造3786 户；实施农村改厕6000 座。

　　8、完成570 个村庄的亮化工程。

　　9、完善34 个街道、65 个乡镇、188 个社区就业信息网络平台。

　　10、提高新农合筹资标准、企业退休职工养老保险待遇；城镇职工医保政策内报销比例80%以上，居民医保门诊报销额度提高到300 元；进一步增加门诊医疗慢性病种类。

　　11、城乡低保、农村五保、重点优抚对象的生活补助标准分别提高10%。

　　12、建设市中小学示范性实践学校；新建、改扩建幼儿园15 所；改造维修城乡校舍10 万平方米。

　　13、建立国民体质检测中心；新增8 套公园健身设施；免费发放篮球1 万个。

　　14、在乡镇建设10 个2000 平方米以上的农资、日用品超市。

　　15、新建、完善农村养老互助幸福院100 个；启动市社会养老院改扩建工程。

　　我们将根据人民群众实际需求，尽力为群众办实事、解难事。

　　（九）深化改革创新，增强发展活力

　　开展“改革创新年”活动，按照国家和省改革总体部署，认真制定我市改革的实施意见，跟上改革步伐，同时更加注重工作创新。

　　进一步深化行政审批制度改革，做好上级下放审批事项的承接工作，继续向县区和市场放权，全面推动网上审批。完成食品药品监督机构职责调整和改革。全面推进事业单位分类改革。

　　清理规范税收优惠政策，实行全市统一政策。着力消除市场壁垒，鼓励引导民间资本进入基础设施、公用事业、保障性住房、商贸流通、养老事业等领域。建立中介机构公平竞争的市场环境、依法依规经营的制度规范。

　　加强政府债务管理，严格执行市本级偿债准备金管理办法。各级政府都要建立偿债准备金制度。

　　加大政府性基金预算和国有资本经营预算公开力度，做好市本级“三公”经费公开，有计划推进部门预算公开。

　　加快国家扶贫改革试验区建设，积极争取国家、省政策支持，探索扶贫改革新模式。继续实施“集团帮村”工程，减少低收入贫困人口5 万人。

　　鼓励农民将承包经营权向专业大户、家庭农场、农民合作社流转，全年新增土地流转30 万亩。扶持农村各类合作经济组织，市级专业合作示范社达到30 家。抓好农村公路改革试点工作。

　　鼓励社会力量和社会资本兴办教育、参与文化产业发展和公共文化服务体系建设。深入推进医药卫生体制改革，做好县级医院综合改革试点工作，继续实施基本药物制度。

　　深化工作创新年活动，围绕产业升级、项目建设、招商引资、企业提升、社会事业发展、民生改善等，创新工作思路举措，突破薄弱环节，破解瓶颈制约，保障要素供给。坚持以效率为中心，促进部门行政审批规范运行、程序简化、运转提速、限时办结。进一步理顺产业园区管理机制，省级以上园区实施封闭运行。

　　（十）加强政府自身建设，着力打造为民务实清廉的政府

　　大力推进改革创新和作风转变，提高行政能力和管理水平，更好地肩负起突破发展的重要使命。

　　建设改革创新、奋发有为的政府。把改革创新贯穿于经济社会发展各个领域、各个环节，体现在政府各项工作中，体现在各项决策中。坚持以思想创新促进工作创新，着力破除“小成即满”思想和惯性思维束缚，突破制约发展的体制机制障碍。坚持高标准确定目标、高效率推进落实、高质量完成工作，体现“ 突破决心”，创建“突破方式”，追求“突破效益”。坚持领导干部率先垂范，打造干事创业的领导班子和干部队伍。

　　建设心系百姓、执政为民的政府。扎实开展群众路线教育实践活动，着力解决“四风”方面存在的问题，切实提高服务群众的能力和水平，倾心尽力为群众办实事、解难事，以实实在在的行动和成效取信于民。优化市、县、乡三级行政服务中心功能，提高窗口单位办事效率和服务水平。抓好政务公开，提高政府工作透明度。完善政府诚信体系，坚决兑现各项承诺，提高政府执行力和公信力。

　　建设真抓实干、高效务实的政府。突出工作重点，把主要精力放在经济发展、社会进步、民生改善上。建立严格的工作责任制，实行领导干部包产业、包项目制度，确保各项工作有目标、有举措、有标准、有时限。坚持雷厉风行的工作作风，亲力亲为推进工作。坚持“ 就事论事”研究工作，推进项目逐个落实、问题逐一解决。改进绩效考核制度，增加资源环境、科技创新、安全生产等指标权重。

　　建设依法行政、清正廉洁的政府。自觉接受市人大、市政协和社会监督，切实提高依法决策、科学决策、民主决策水平。巩固规范管理年活动成果，坚持用制度管权管事管人，推动各级干部“ 讲诚信、懂规矩、守纪律”。加强软环境建设，开展“浮庸散奢”专项治理。继续推进“五大系统”建设，有效发挥公共资源交易平台作用。贯彻落实《党政机关厉行节约反对浪费条例》，严格经费预算，控制“三公”支出，降低行政成本，建设节约型机关。进一步强化领导干部经济责任审计监督，加强国有资产监管，严肃查处违法违纪案件，努力打造干部清正、政府清廉、政治清明的良好局面。

　　各位代表，我市的突破发展任务艰巨，使命光荣。让我们在市委的领导下，把握发展机遇，坚持改革创新，推进突破发展，全面完成各项目标任务，努力开创转型振兴的新局面！
